# Telocytes/CD34+ Stromal Cells in Pathologically Affected White Adipose Tissue

**DOI:** 10.3390/ijms21249694

**Published:** 2020-12-18

**Authors:** Lucio Díaz-Flores, Ricardo Gutiérrez, Ma Pino García, Miriam González-Gómez, Jose Luís Carrasco, Hugo Alvarez-Argüelles, Lucio Díaz-Flores

**Affiliations:** 1 Department of Basic Medical Sciences, Faculty of Medicine, University of La Laguna, 3071 Tenerife, Spain; histologia54@gmail.com (R.G.); mirgon@ull.edu.es (M.G.-G.); jcarraju@ull.edu.es (J.L.C.); hargue@ull.es (H.A.-A.); ldfvmri@yahoo.es (L.D.-F.J.); 2 Department of Pathology, Eurofins® Megalab–Hospiten Hospitals, 38100 Tenerife, Spain; mpgarcias@megalab.es

**Keywords:** telocytes, CD34+ stromal cells, adipose tissue, repair, tumour stroma, adipose tissue tumours, amyloidosis, irradiation

## Abstract

We studied telocytes/CD34+ stromal cells (TCs/CD34+SCs) in pathologically affected white adipose tissue after briefly examining them in normal fat. To this aim, we reviewed pathological processes, including original contributions, in which TCs/CD34+SCs are conserved, increased, and lost, or acquire a specific arrangement. The pathologic processes in which TCs/CD34+SCs are studied in adipose tissue include inflammation and repair through granulation tissue, iatrogenic insulin-amyloid type amyloidosis, non-adipose tissue components (nerve fascicles and fibres in neuromas and hyperplastic neurogenic processes) and tumours (signet ring carcinoma with Krukenberg tumour and colon carcinoma) growing in adipose tissue, adipose tissue tumours (spindle cell lipoma, dendritic fibromyxolipoma, pleomorphic lipoma, infiltrating angiolipoma of skeletal muscle and elastofibrolipoma), lipomatous hypertrophy of the interatrial septum, nevus lipomatosus cutaneous superficialis of Hoffman–Zurhelle and irradiated adipose tissue of the perirectal and thymic regions. Two highly interesting issues emerged: (1) whether the loss of CD34 expression in TCs/CD34+SCs is by changes in marker expression or the disappearance of these cells (the findings suggest the first possibility) and (2) whether in some invasive and metastatic malignant tumours, TCs/CD34+SCs that completely surround neoplastic cells act as nurse and/or isolating cells. Further studies are required on adipose tissue TCs/CD34+SCs, mainly in lipomatosis and obesity.

## 1. Introduction

White adipose tissue is formed by unilocular adipocytes and the extracellular vascular fraction, which includes endothelial cells of both blood and lymphatic vessels, pericytes, Schwann cells of peripheral nerves, fibroblasts, macrophages and telocytes/CD34+ stromal cells (TCs/CD34+SCs). Adipose tissue participates in (a) fat storage (triglycerides) (energy storage), (b) the production of hormones (e.g., lectin, adiponectin and resistin), growth factors and cytokines, (c) thermal regulation, mainly subcutaneous fact and (d) support and mechanical protection (lower effect of impacts on organs). Other “non-traditional” functions, widely reviewed in different locations by Zwick et al. (2017) [1], include (a) the modulation of tissue growth and regeneration by paracrine signals (e.g., progenitor signalling to hair follicles, mammary gland and bone marrow cell components), (b) participation in repair and cancer by crosstalk signalling and (c) the contribution to innate immunity in the skin and intestine by cytokines and antimicrobial peptides. In addition, human adipose tissue is an abundant and easily procured source of CD34+ adipose-derived stromal cells (ASCs) for studies in vitro and tissue engineering, which have received great attention [2,3,4,5].

Telocytes are a type of stromal cell. Under electron microscopy and in normal conditions, they show a small somatic body and two or several long, slender, moniliform cytoplasmic processes (telopodes) with podomeres (thin segments) and podoms (dilated portions) [6,7]. Since their discovery in 2010 [7], telocytes have been described in the interstitium of many tissues, including adipose tissue. However, no review has focused on studying the current state of telocyte behaviour in adipose tissue affected by pathologic processes. Telocytes express CD34, among other markers, and therefore correspond to the CD34+ stromal cells observed in light microscopy [8,9,10,11,12,13,14,15,16,17]. This immunohistochemical expression of telocytes, which allows us to consider them as telocytes/CD34+stromal cells (TCs/CD34+SCs), facilitates the correlation with previous descriptions or original observations of CD34+ resident/native stromal/interstitial cells in pathologic adipose tissue.

Given the above, the objective of this work was to review the current stage of TCs/CD34+SCs in adipose tissue, mainly in pathologic conditions. In the first section, we briefly describe the characteristics of these cells in normal adipose tissue. In the following sections, we consider TC/CD34+SC behaviour in adipose tissue affected by pathologic processes, including inflammation and repair through granulation tissue, iatrogenic insulin-amyloid type amyloidosis, non-adipose tissue components and tumours growing in adipose tissue, adipose tissue tumours and other processes, such as the lipomatous hypertrophy of the interatrial septum, nevus lipomatosus cutaneous superficialis of Hoffman–Zurhelle and irradiated adipose tissue. Finally, we give a general overview of the response of TCs/CD34+SCs in the different conditions observed and propose future studies on these cells in adipose tissue.

## 2. TCs/CD34+SCs in Normal Adipose Tissue

**Figure 1:**
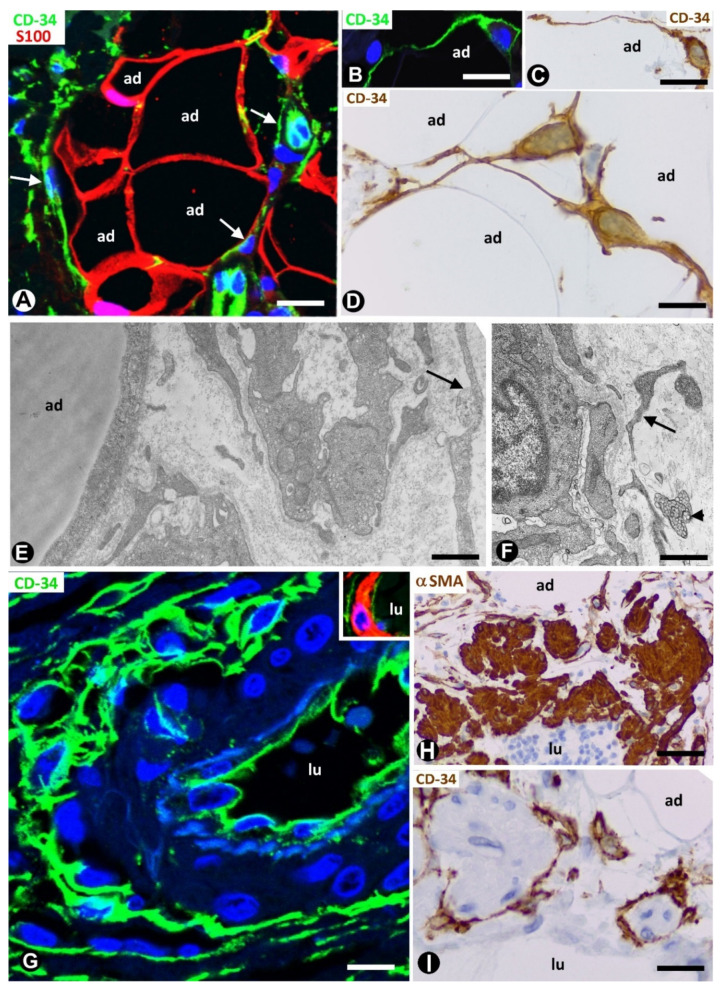
Telocytes/CD34+ stromal cells (TCs/CD34+SCs) in the normal adipose tissue. (**A**): TCs/CD34+SCs (green, arrows) in a septum and in a lobular region, in which adipocytes (ad) express S100 protein (red). (**B**–**D**): TCs/CD34+SCs (green in (**B**), and brown in (**C**,**D**) with small somatic bodies and long, thin processes between adipocytes (ad). (**E**,**F**): telopodes of telocytes (arrows) are ultrastructurally observed around small vessels. Note in F an extracellular multivesicular body (arrowhead) next to the telopodes. G and insert: a large septal vessel showing a double-ring appearance with anti-CD34 staining (green). Note that CD34 stained ECs in the intima and TCs/CD34+SCs in the adventitia form two stained circles “sandwiching” the unstained (**G**) or red αSMA stained (Insert) media layer. (**H**,**I**): lymphatic collectors in which parietal smooth muscle cells (expressing αSMA, (**H**)) are surrounded by TCs/CD34+SCs (**I**). Lumen = lu in blood (**G**) and lymphatic (**H**,**I**) vessels. A: double immunofluorescence labelling for CD34 (green) and protein S100 (red). B and G: immunofluorescence labelling for CD34 (green). (**C**,**D**,**I**): immunochemistry for CD34. E and F: ultrathin sections. Uranyl acetate and lead citrate. Insert of G: double immunofluorescence staining for CD34 (green) and αSMA (red). H: immunochemistry for αSMA (brown). DAPI (4′,6-diamidino-2-phenylindole) (blue) counterstain for nuclei in A, B, G and insert of G, and haematoxylin counterstain in (**C**,**D**,**H**,**I**). Bar: (**A**,**H**,**I**): 50 µm; (**B**,**C**,**G**): 30 µm; (**D**): 20 µm; (**E**,**F**): 4 µm.

In adipose tissue, TCs/CD34+SCs are widely distributed in the septa and to a lesser extent in intralobular regions (Figure 1A). This distribution is important because different progenitor roles have been attributed to these cells, depending on their septal or intralobular location [18] (see below). In intralobular regions, TCs/CD34+SCs show a small somatic body and long, thin processes, which extend alongside adipocytes (Figure 1B–D) and the microvasculature (Figure 1E,F). Extracellular multivesicular bodies are present near telopodes (Figure 1F), a finding previously described in other tissues, with functional importance in intercellular communication [19] and in accordance with the hypothesis about the role of TCs/CD34+SCs in regulating the local microenvironment by cell-to-cell contacts and extracellular shedding vesicles [6,9,10,20,21,22,23]. In the septa, these cells form a network, which surrounds differently sized vessels (location in the vessel adventitia) and between collagen fibres. Thus, when stained with anti-CD34, the vessels show a double-ring appearance with two CD34-stained concentric circles “sandwiching” the unstained media layer (Figure 1G). Indeed, by double staining with anti-CD34 and anti-αSMA (α smooth muscle actin), the blood vessels in the adipose tissue present CD34+ αSMA- endothelial cells (ECs) in the intima layer, CD34- αSMA+ pericytes/vascular smooth muscle cells in the media layer and CD34+ αSMA- stromal cells in the external layer (insert in Figure 1G). The long, thin processes of the TCs/CD34+SCs in the external layer of these vessels also extend into the collagenous tissue of the septa. The small lymphatic vessels in adipose tissue present TCs/CD34+SCs around ECs. TCs/CD34+SCs are also observed surrounding small groups of smooth muscle cells in the pre-collector and collector lymphatic vessels (Figure 1H,I).

## 3. TCs/CD34+SCs in Pathologically Affected Adipose Tissue

### 3.1. TCs/CD34+SCs of Adipose Tissue in Early and Most Advanced Stages of Inflammation and Repair through Granulation Tissue

**Figure 2:**
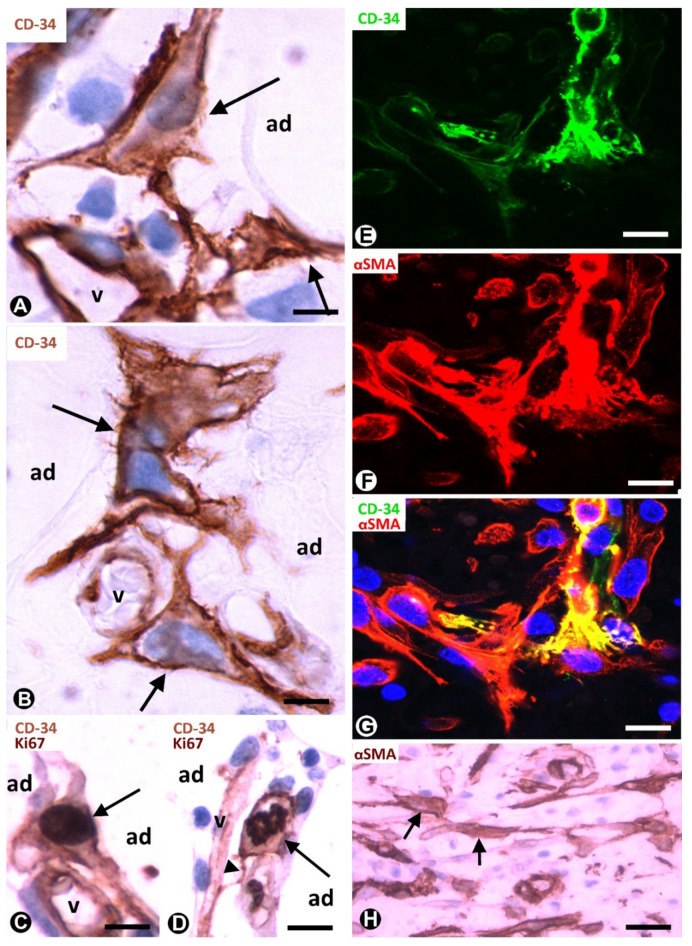
TCs/CD34+SCs of the adipose tissue in early (**A**–**G**) and most advanced (**H**) stages of repair through granulation tissue. (**A**,**B**): bulky TCs/CD34+SCs (arrows) with enlarged nuclei are observed around small vessels (v) and adipocytes (ad). (**C**): the co-expression of CD34 and Ki-67 in a TC/CD34+SC (CD34 express in the membrane and in the periphery of cytoplasm, and Ki-67 in the nucleus) (arrow). (**D**): the co-expression of CD34 (in the membrane and peripheral cytoplasm) and Ki-67 (in chromosomes) is shown in a TC/CD34+SC in mitosis (arrow). Note in this cell a thin process (telopode, arrowhead) in contact with a capillary (v). (**E**–**G**) the co-expression of CD34 (green) and αSMA (red) in stromal cells. H: stromal cells expressing αSMA are present in an advanced stage of repair. A and B: immunochemistry for CD34. (**C**,**D**): sections are doubly immunostained with CD34 and Ki-67 (CD34 stained the cell membrane and peripheral cytoplasm, and Ki-67 the nucleus). (**E**–**G**): confocal microscopy, frontal view, immunofluorescent label with anti-CD34 (green) and anti-αSMA (red). (**H**): immunochemistry for αSMA. Haematoxylin contrast in (**A**–**D**,**H**). DAPI (blue) counterstain in G. Bar: (**A**,**B**): 10 µm; (**C**–**G**): 20 µm; (**H**): 40 µm.

Native (resident) TCs/CD34+SCs lose their CD34 expression during inflammatory/repair processes in adipose tissue, while numerous cells expressing αSMA are seen in the affected tissue region [24,25]. Successive culture passages of CD34+ cells that form part of the freshly isolated stromal fraction obtained from adipose tissue give a similar finding. Indeed, the CD34 expression of these cells only occurs during early passages [2,3,4,5]. In successive passages, the loss of CD34 expression is followed by the acquisition of other markers, depending on culture conditions [2,3,4,5]. Thus, CD34+ cells adhere to culture plastic, proliferate and differentiate in several tissue components, including adipocytes, chondrocytes, osteoblasts and myocytes. αSMA is among the new markers and when it is expressed, the cells acquire a myofibroblastic-like aspect. In human adipose tissue with inflammatory/repair processes (peri-appendicitis, peri-diverticulitis and actinomycosis with localised peritoneal abscesses), the number, morphology and expression of the markers in the stromal cells depend on the evolutionary stage and the distance from the lesion location. Thus, in early stages, TCs/CD34+SCs increase the somatic volume and size of the nucleus (Figure 2A,B), which present one or two prominent nucleoli. Frequent mitoses and a high proliferative index are seen in these cells, which show the co-expression of CD34 and Ki-67 (Figure 2C,D). Subsequently, the co-expression of CD34 and αSMA can be observed in some stromal cells (Figure 2E–G). In the most advanced stages, the stromal cells express αSMA (Figure 2H). Therefore, resident TCs/CD34+SCs in adipose tissue may be a source of stromal αSMA+ cells with a myofibroblastic aspect. As for the influence of the distance from the lesion location, it has been demonstrated that the increased number and size of activated stromal cells close to actinomycotic abscesses show αSMA expression, while those further away retain their CD34 expression [24,26].

Repair includes two types of processes: regeneration and repair through granulation tissue. The participation of TCs/CD34+SCs in regeneration has been well studied in several tissues and organs [25,27,28,29,30,31,32,33,34,35,36,37,38,39,40], while the role of these cells in repair through granulation tissue has received less attention [24,25]. In this provisional tissue, myofibroblasts are a main component, with associated macrophages and numerous small blood vessels. Granulation tissue may remain as such (mainly in the stroma of certain tumours, see below) or may be followed by (a) fibrous tissue, with scarring or organisation (formation of new masses of fibrous tissue in blood clots or inflammatory exudates with fibrin deposits) and (b) other tissues, such as cartilage and bone. Adipose tissue, plastic, abundant and rich in CD34+ resident stromal cells, is therefore highly suitable for correlating the behaviour of TCs/CD34+SCs in in vivo and in vitro studies and for following the changes of these cells in early stages of repair to granulation tissue. Thus, the contributions in this section confirm the previous observations of our group supporting the hypothesis of TCs/CD34+SCs as precursor cells [24,25].

### 3.2. TCs/CD34+SCs in Adipose Tissue with Iatrogenic Insulin-Amyloid Type Amyloidosis

A particularly good example of TCs/CD34+SCs in the adipose tissue pathology of endocrine origin is the involvement of subcutaneous adipose tissue in iatrogenic insulin-amyloid type amyloidosis. Indeed, insulin-derived amyloidosis can occur in adipose tissue at sites of repeated insulin administration (insulin injection amyloidosis, iatrogenic insulin-amyloid type, injected-localised insulin amyloid, insulin balls, amyloidoma).

**Figure 3:**
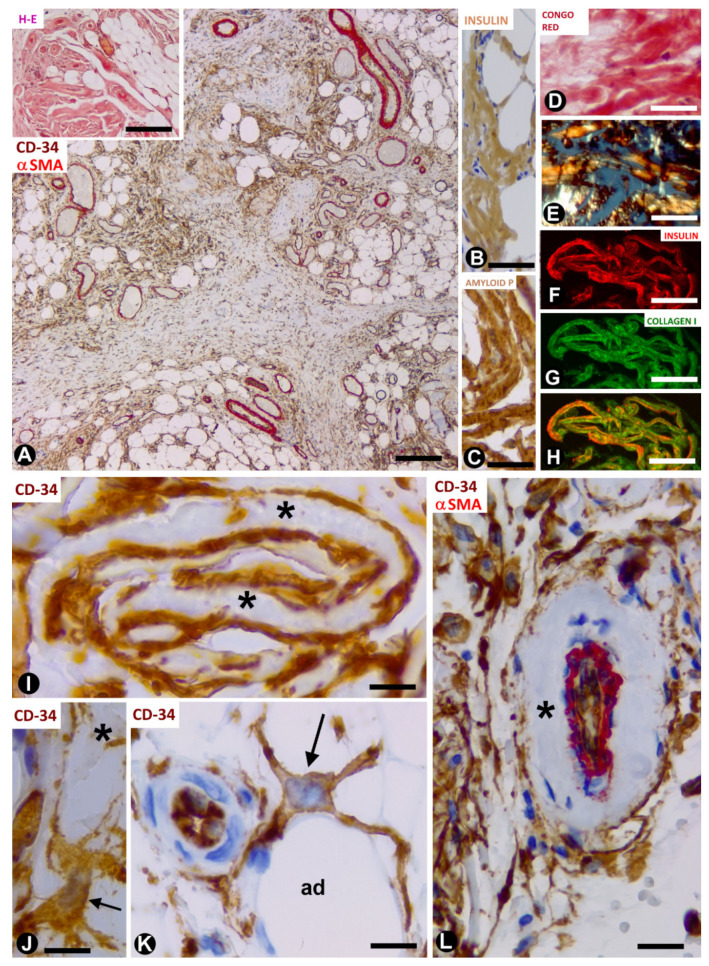
TCs/CD34+SCs in adipose tissue with iatrogenic insulin-amyloid type amyloidosis. (**A**): numerous TCs/CD34+SCs in adipose lobules are arranged between fibrous tracts. Double immunochemistry labelling for CD34 (brown) and αSMA (red). Insert: a nodule with haematoxylin staining. (**B**–**H**): amyloid deposits showing immunochemistry labelling for insulin (**B**) and amyloid P (**C**), Congo red positivity (**D**) with yellow-green birefringence under polarised light (**E**), and the colocalisation of insulin and collagen (**I)** ((**F**): insulin—red; (**G**): collagen (**I**)—green and (**H**): the colocation of both). (**I**): projections of TCs/CD34+SCs (brown) around folded amyloid deposits (asterisks). (**J**–**L**): TCs/CD34+SCs (brown, arrows) with projections around an amyloid deposit ((**J**), asterisk), adipocytes ((**K**), ad) and amyloid components in the adventitia (asterisk) of a vessel with double immunochemistry labelling for CD34 (brown) and αSMA (red). Bar: ((**A**) and insert): 160 µm; (**B**–**H**): 50 µm; (**I**–**K**): 20 µm; (**L**): 60 µm.

In a study of this rare process using morphological and immunohistochemical procedures, we observed numerous TCs/CD34+SCs in differently sized amyloidotic nodules in subcutaneous adipose tissue (Figure 3A) (unpublished observations). TCs/CD34+SCs are present around thick deposits (with a sheet aspect) containing collagen and amyloid (see below) and vessels in the amyloid nodules, especially in the smallest. TC telopodes surrounding amyloid deposits have been described in the human heart (isolated atrial amyloidosis) [41]. Amyloidosis in the heart differs from insulin-derived amyloidosis because of the association of amyloid material with collagen in the latter. Then, we will examine amyloid deposits, and the behaviour of TCs/CD34+SCs in amyloidogenic nodules in adipose tissue.

In the nodules (Figure 3A, insert), the amyloid deposits are located near adipocytes (Figure 3A) and show immunostaining for anti-insulin (Figure 3B) and amyloid P (Figure 3C), as well as Congo red positivity (Figure 3D), with yellow-green birefringence (Figure 3E). The amyloid deposits are negative for anti-A-amyloid and anti-transthyretin. Under confocal microscopy, the colocalisation of collagen I and insulin is demonstrated in the amyloid deposits (Figure 3F–H). TCs/CD34+SCs are increased in number and size, and are arranged in close association with these extracellular deposits formed by amyloid and collagen I. Indeed, TCs/CD34+SCs show ovoid or elongated nuclei and bipolar or multipolar cytoplasmic processes (telopodes), which surround the amyloid deposits (Figure 3I,J) and adipocytes (Figure 3K). Telopodes encircling the amyloid deposits can be related to the role of TCs in organising and controlling the extracellular matrix. Thus, the ability of these cells to limit amyloid spread has been suggested in atrial amyloidosis [41]. TCs/CD34+SCs are negative for anti-CD31, anti-CD45, anti-αSMA and anti-h-caldesmon. In the vessels, TCs/CD34+SCs are also located around amyloid and collagenous materials, which are deposited in the transition between the media layer and the adventitia (Figure 3L). In the central areas of the largest nodules, in which amyloid and collagenous deposits converge, the number of adipocytes, vessels and TCs/CD34+SCs decreases dramatically. Insulin amyloid has toxic action [42] and the decline in adipocytes, vessels and TCs/CD34+SCs in the centre of large nodules may be due to this action. Isolated CD68+ macrophages are present, some with a signet ring aspect. The association of TCs/CD34+SCs and macrophages is also observed (the last two observations are shown). In this section, we therefore contribute the presence of TCs/CD34+SCs (in high numbers) in insulin-derived amyloid type amyloidosis, and their possible role as regulators of local homeostasis.

### 3.3. TCs/CD34+SCs in Non-Adipose Pathologic Processes Growing in Adipose Tissue

In this section, we present examples of adipose tissue infiltration by pathologic processes in which resident TCs/CD34+SCs behave differently. These processes include neuromas and neurogenic hyperplasia, signet ring carcinoma with Krukenberg tumour and peritoneal dissemination, and colon adenocarcinoma.

#### 3.3.1. TCs/CD34+SCs in Neuromas and Hyperplastic Neurogenic Processes Affecting Adipose Tissue

**Figure 4:**
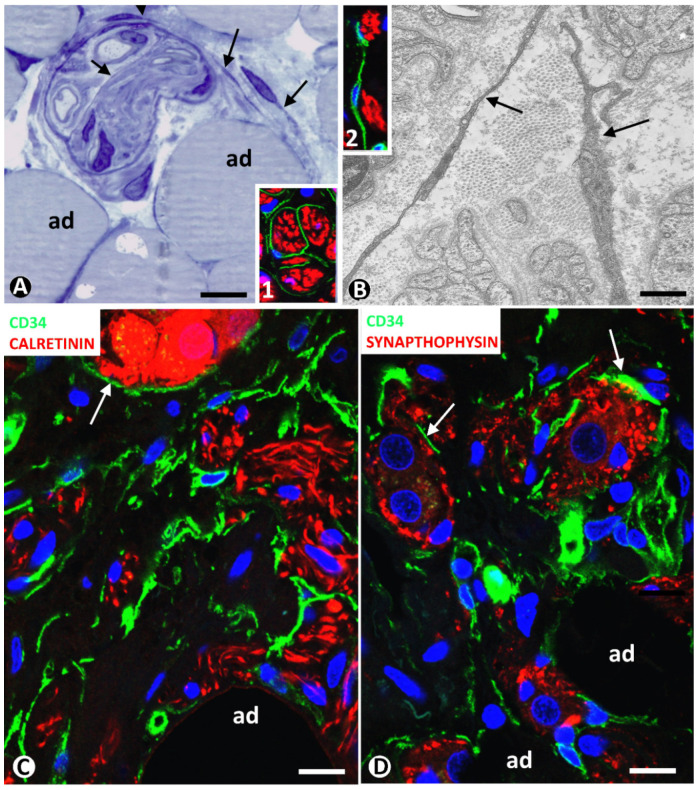
(**A**,**B**) TCs/CD34+SCs in neuromas. (**A**): a nerve fascicle between adipocytes (ad), TCs (arrows) are observed around and within the fascicle in a semithin section stained with toluidine blue. (**B**): ultrastructural image showing TC telopodes (arrows) around nerve fibres. Ultrathin section. Uranyl acetate and lead citrate. Inserts in (**A**,**B**), TCs/CD34+SCs (green) and Schwann cells (red) shown by double immunofluorescence labelling for CD34 (green) and S100 protein (red). (**C**,**D**): TCs/CD34+SCs (green) around nerve fibres and neuro-glial units (arrows) (red) between adipocytes (ad) in a hyperplastic neurogenic process. Double immunofluorescence labelling for CD34 (green) and calretinin ((**C**), red) or synaptophysin ((**D**), red). Bar: (**A**,**C**,**D**): 40 µm; (**B**): 4 µm; inserts of (**A**) and (**B**): 50 µm.

Neuromas can extend to adipose tissue, with the presence of nerve fascicles and isolated nerve fibres between adipocytes. These nervous components in adipose tissue can be very numerous. Using immunofluorescence in confocal microscopy, and semithin and ultrathin sections, we observe TCs/CD34+SCs around and within nerve fascicles (Figure 4A,B and inserts). Telopodes of these cells were seen near nerve fibres (Figure 4A,B and inserts 1 and 2).

In a previous review on TCs/CD34+SCs in the peripheral nervous system, we highlighted the behaviour of these cells in neuropathies of the appendix and gallbladder [43]. Using confocal microscopy, we then studied the involvement of adipose tissue in appendicular hyperplastic neurogenic processes. TCs/CD34+SCs and their telopodes were observed around nerve fibres and their accompanying neurons (neural–glial units) in adipose tissue (Figure 4C,D). Neuromas and hyperplastic neurogenic processes are therefore examples of TC/CD34+SC activation conserving CD34 expression.

#### 3.3.2. CD34+ and αSMA+SCs in Tumours Infiltrating Adipose Tissue

We chose two types of infiltrating malignant epithelial neoplasms in adipose tissue as examples of the different behaviour of tissue resident TCs/CD34+SCs: with the conservation or loss of CD34 expression, and with the gain of αSMA expression. Signet ring cell carcinoma with Krukenberg tumour and peritoneal dissemination, and colon adenocarcinoma are taken into consideration below.

**Figure 5:**
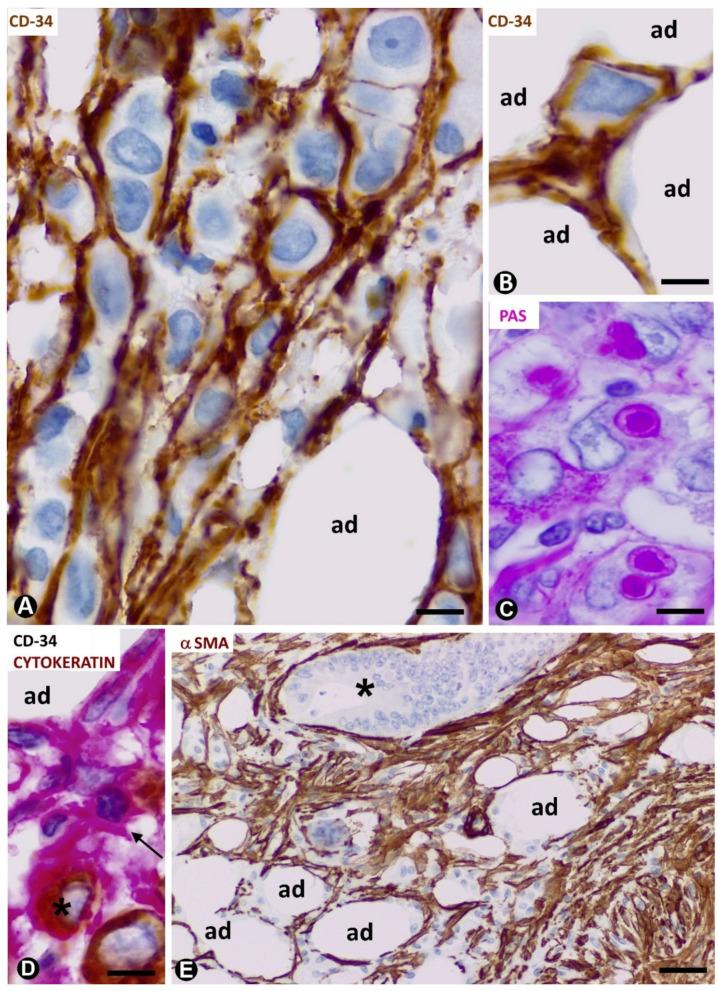
CD34+ and αSMA+ stromal cells in tumours infiltrating adipose tissue. (**A**–**D**): signet ring carcinoma with Krukenberg tumour and peritoneal dissemination. TCs/CD34+SCs are observed around neoplastic cells (**A**,**B**,**D**), which show PAS+ vacuoles (**C**) and express cytokeratin AE1/AE3 (**D**). (**A**,**B**): CD34 immunochemistry; haematoxylin counterstain. (**C**): PAS staining. (**D**): double immunochemistry labelling for CD34 (red) and cytokeratin AE1/AE3 (brown). (**E**): neoplastic glands (asterisk) of adenocarcinoma of colon infiltrating adipose tissue. αSMA cells (brown) are observed in the stroma. αSMA immunochemistry. Haematoxylin counterstain. Bar: (**A**–**D**): 20 µm; (**E**): 100 µm.

We observed an important increase in adipose resident TCs/CD34+SCs in signet ring cell carcinoma with Krukenberg tumour and peritoneal dissemination (Figure 5A) (original observation). A striking finding is that TCs/CD34+SCs completely surround each cell or groups of two or three neoplastic cells (Figure 5A,B,D) (unpublished observation), which show intracytoplasmic PAS (Periodic acid–Schiff) positive vacuoles (Figure 5C) and cytokeratin expression (Figure 5D). Further research is required to clarify whether this encircling action represents a nurse cell behaviour, tumour cell isolation or both (see below).

All stromal cells around neoplastic glands in the adipose tissue infiltrated by adenocarcinoma of the colon show αSMA expression (Figure 5E). The changes from TCs/CD34+SCs to stromal cells expressing αSMA in this type of advanced neoplasm cannot be well demonstrated, unlike in the early stages of repair through granulation tissue, in which follow up is possible.

## 4. TCs/CD34+SCs in Tumours/Pseudo-Tumours of Adipose Tissue

Adipose stromal cells can participate in the development of lipomas, which contain CD34+ cells [44]. In addition, these cells isolated from lipomas show strong adipogenic potential and have been proposed for regenerative medicine and tissue engineering [45,46,47,48]. In this section, we present examples of benign tumours/pseudo-tumours of adipose tissue in which a greater number of TCs/CD34+SCs are observed, including spindle cell lipoma, dendritic fibromyxolipoma, pleomorphic lipoma, infiltrating angiolipoma of skeletal muscle and elastofibrolipoma. In addition to confirming the presence of a high population of TCs/CD34+SCs in these tumours, we suggest that these cells can be a source of the myxoid component present in some of them.

### 4.1. TCs/CD34+SCs in Spindle Cell Lipoma

**Figure 6:**
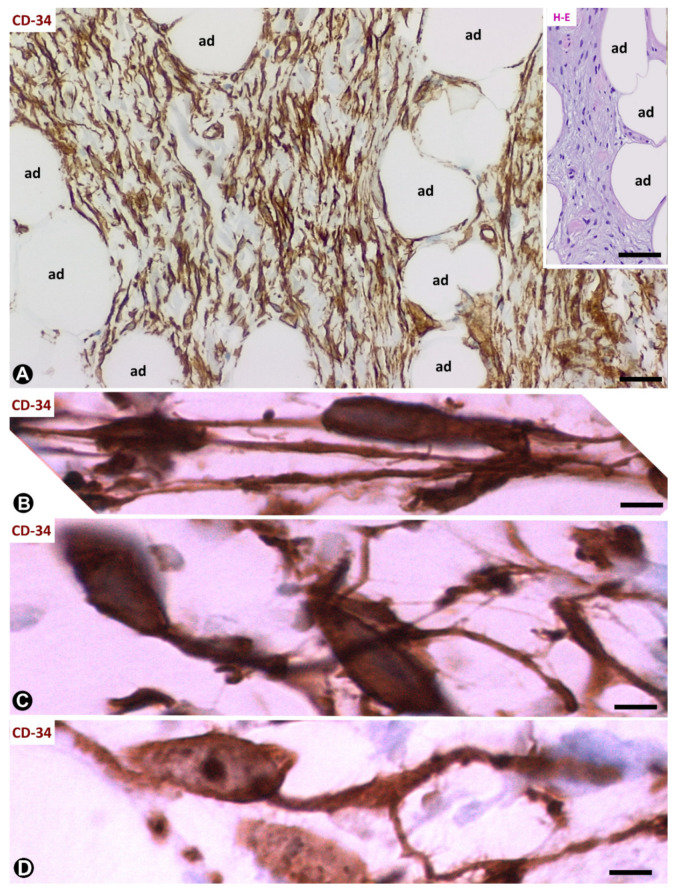
Spindle cell lipoma. (**A**): CD34+ spindle cells (brown) are observed between adipose cells (ad). Insert shows an image with haematoxylin–eosin (HE) staining in which it is difficult to distinguish processes from spindle cells. (**B**–**D**): CD34+ spindle cells with bipolar processes (**B**), piriform aspect with a single (**C**) and branched (**D**) process. (**A**–**D**): CD34 immunochemistry; haematoxylin counterstain. Bar: (**A**): 80 µm; (insert of (**A**)): 100 µm; (**B**–**D**): 10 µm.

In spindle cell lipoma, a benign tumour described in 1975 [49], TCs/CD34+SCs and mature adipose tissue are the main components (Figure 6A). The proportion of each component varies considerably depending on the case and even within the same case. TCs/CD34+SCs show ovoid nuclei with round ends. These cells strongly express CD34 and negativity for S100 protein, desmin and αSMA. Though poorly defined with haematoxylin–eosin (HE) staining, when immunostained with anti-CD34, the cells present a somatic region and thin processes, which are generally bipolar (Figure 6A,B) and less frequently multipolar. On occasions, the somatic region of TCs/CD34+SCs acquires a piriform aspect, a single process emerging from it (Figure 6C). Branched processes are also observed (Figure 6D).

TCs/CD34+SCs are associated with (a) mature adipocytes (absence of lipoblasts in the tumour; an important finding in the tumour differential diagnosis); (b) thick collagen bundles (in some tumours with abundant fibrous tissue); (c) a prominent vasculature (occasionally hyalinised) and (d) scattered mast cells or groups of lymphocytes.

### 4.2. TCs/CD34+SCs in Dendritic Fibromyxolipoma

**Figure 7:**
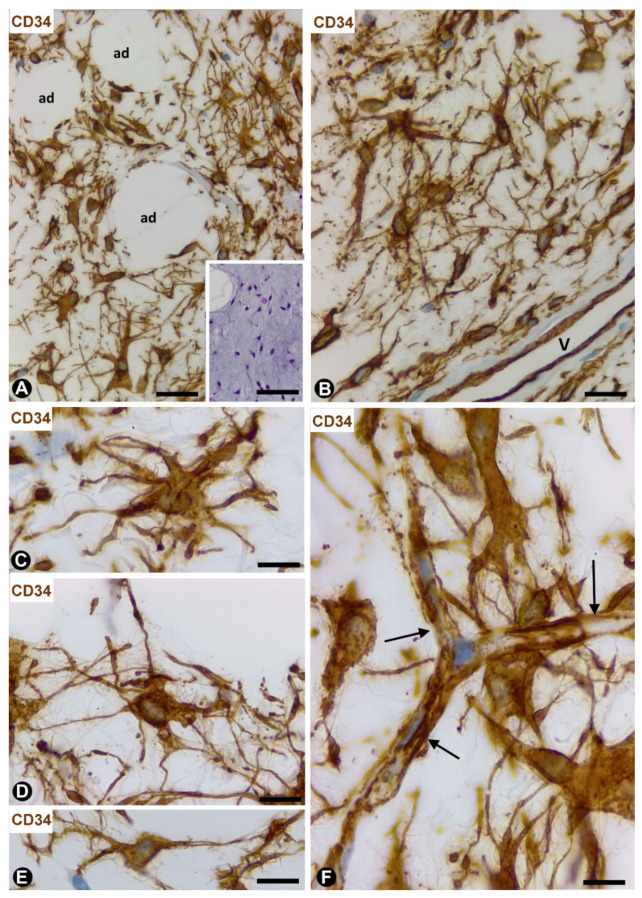
Dendritic fibromyxolipoma. (**A**–**F**): CD34 immunochemistry with haematoxylin counterstain. CD34+ dendritic cells (brown) between adipocytes (ad) (**A**) and near a vessel (v, (**B**)). Observe the stellate aspect (**C**,**D**) and contacts with neighbouring cells (**E**) and small vessels ((**F**), arrows). Insert of A: image in a section stained with haematoxylin and eosin, bar: (**A**,**B**): 60 µm; (insert of (**A**)): 100 µm; (**C**–**E**): 20 µm; (**F**): 30 µm.

In dendritic fibromyxolipoma [50], loosely arranged TCs/CD34+SCs in the lesion are predominantly stellate (Figure 7A,B). As in spindle cell lipoma, these cells strongly express CD34 and negativity for S100 protein, desmin and αSMA. TCs/CD34+SCs show a small somatic region in which a triangular or ovoid nucleus is observed. Numerous elongated thin processes extend from the somatic region in the interstitium, the cells acquiring their characteristic dendritic aspect (Figure 7C,D). The processes established contact with other TCs/CD34+SCs (Figure 7E), and/or with thin walled vessels (Figure 7F) (intercellular communication), and partially surround adipocytes (Figure 7A,B). Atypia, necrosis, mitotic activity or lipoblasts are not found.

### 4.3. TCs/CD34+SCs in Pleomorphic Lipoma

**Figure 8:**
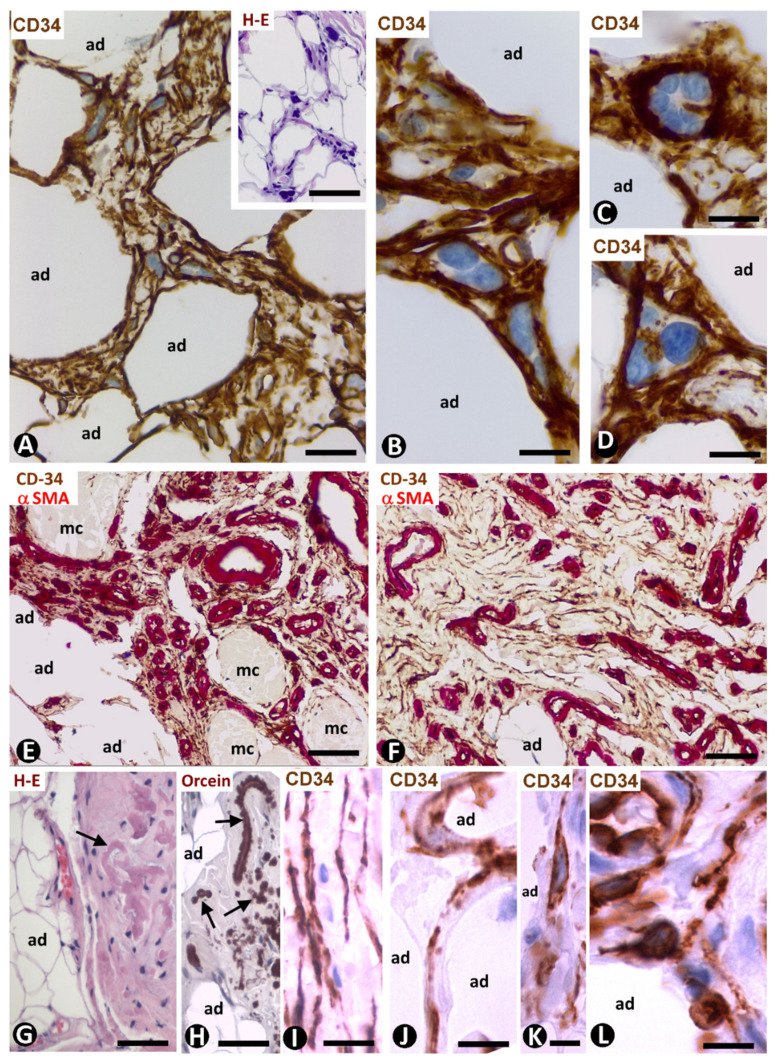
TCs/CD34+SCs in pleomorphic lipoma, infiltrating the angiolipoma of skeletal muscle and elastofibrolipoma. (**A**–**D**): CD34 immunochemistry with haematoxylin counterstain. Pleomorphic lipoma showing CD34+ cells with strong cytoplasmic labelling, intricate processes and pleomorphic nuclei. Note some multinucleated cells. Insert of A: image of the lesion with HE staining; adipocytes (ad). (**E**,**F**): double immunochemistry for CD34 (brown) and αSMA (red). Numerous spindle CD34+ cells (brown) in the infiltrating angiolipoma of skeletal muscle with abundant microvessels (periendothelial αSMA+ cells—red) between skeletal muscle cells (mc) and adipocytes (ad). (**G**–**L**): elastofibrolipoma by HE staining (**G**), orcein staining (**H**) and with immunochemistry for CD34 (**I**–**L**). The fibrous, adipose and elastic components are shown in (**G**,**H**). CD34+ cells are observed in the fibrous tracts (**I**) and between adipocytes (**J**–**L**). Bar: (**A**): 40 µm; (insert of **A**): 100 µm; (**B**–**D**) 20 µm; (**E**–**H**): 60 µm; (**I**–**L**): 30 µm.

The relationship between spindle cell lipoma and pleomorphic lipoma was already considered in early descriptions of the pleomorphic lipoma, under this name [51] or other terms, such as polymorphous or ancient spindle cell lipoma. Though currently considered variants of a single entity, the characteristics of TCs/CD34+SCs in this pleomorphic and generally circumscribed benign lesion warrant special attention. Thus, variable numbers of spindle, pleomorphic and multinucleated giant cells with a floret-like aspect are observed, all showing strong positivity for CD34 (Figure 8A–D) and negativity for CD68, S100, αSMA, desmin and Ki-67. The nuclei are hyperchromatic and frequently lobulated. Numerous processes of these cells form a complex labyrinthine system in which it is difficult to identify each process independently when immunostained with anti-CD34 (Figure 8A–D). The cells are located between a fibrous or myxoid stroma, with numerous thick short fragments of rope-like collagen, mature adipocytes (lipoblasts are absent), vessels and some mast cells.

### 4.4. TCs/CD34+SCs in Infiltrating Angiolipoma of Skeletal Muscle

Numerous TCs/CD34+SCs are observed in the interstitium between vessels, adipocytes and myocytes in infiltrating the angiolipoma of muscle (Figure 8E,F). This finding occurs in all three types of the lesion (small vessel, large vessel and mixed types). Generally, TCs/CD34+SCs are spindle-shaped with two large, thin bipolar processes, which extend around collagen fibres.

### 4.5. TCs/CD34+SCs in the Elastofibrolipoma

This lesion, formed by elastic, fibrous, and adipose tissues (Figure 8G,H) shows frequent TCs/CD34+SCs (Figure 8I–L). These cells are arranged between mature adipose cells, around vessels and collagen bands of sclerotic connective tissue, as well as near abnormal elastic fibres displaying a bead or globular structure. TCs/CD34+SCs in the lesion are bulky and show variable numbers of processes, which extend between the aforementioned structures (Figure 8I–L).

## 5. TCs/CD34+SCs in Other Processes Affecting Adipose Tissue

### 5.1. TCs/CD34+SCs in Lipomatous Hypertrophy of the Interatrial Septum

**Figure 9:**
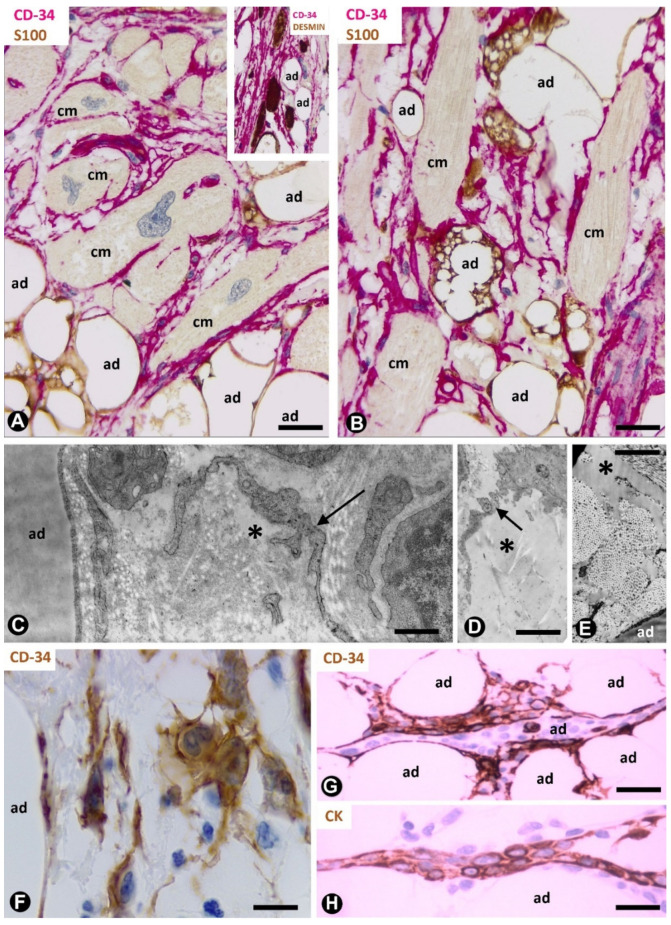
TCs/CD34+SCs in the lipomatous hypertrophy of the interatrial septum, nevus cutaneous superficialis of Hoffman–Zurhelle and irradiated adipose tissue. (**A**,**B**): lipomatous hypertrophy of the interatrial septum. Double immunochemistry labelling for CD34 (red) and S100 protein (brown). Numerous TCs/CD34+SCs (red) are observed between cardiomyocytes (cm) and adipocytes (ad, brown). Note some multivacuolated cardiomyocytes. Insert of A: double immunochemistry labelling for CD34 (red) and desmin (brown). Desmin-positive cardiomyocytes (brown) are surrounded by TCs/CD34+SCs (red); adipocytes: ad. (**C**–**E**): nevus lipomatosus cutaneous superficialis of Hoffman–Zurhelle. Ultrastructural images of a TC ((**D**), arrow) or TC processes ((**C**), arrows) and free fat in the interstitium ((**D**,**E**), asterisks); adipocytes = ad. (**F**–**H**): irradiated fat in rectal and perirectal tissues (**F**) and in the thymic region (**G**,**H**). Immunochemistry labelling for CD34 (**F**,**G**) and cytokeratin (**H**). Increased numbers of TCs/CD34+SCs (brown) showing prominent processes. Observe some of these cells around inflammatory cells (**F**) and strips of thymic epithelium (**G**), which express cytokeratin ((**H**), brown). Bar: (**A**,**B**): 40 µm; (**C**–**E**): 4 µm; (**F**): 20 µm; (**G**,**H**): 40 µm.

Numerous TCs/CD34+SCs are present in the lipomatous hypertrophy of the cardiac interatrial septum (Figure 9A,B) (unpublished observation). This rare lesion shows abundant unilocular and some multilocular adipose cells that infiltrate between cardiomyocytes of the septum [52]. TCs/CD34+SCs are seen surrounding cardiomyocytes and extending between adipocytes (Figure 9A,B). The cardiomyocytes are hypertrophic, degenerative or atrophic and present bizarre nuclei without mitoses (Figure 9A). The surrounding TCs/CD34+SCs form an intricate network, which increases focally.

### 5.2. TCs/CD34+SCs in the Nevus Lipomatosus Cutaneous Superficialis of Hoffman–Zurhelle

The nevus lipomatosus superficialis of Hoffman–Zurhelle can be considered as an ectopic adipose tissue in the dermis or hamartoma-like lesion, in which grouped or dispersed fat lobules are located between collagenous tissue. The adipose lobules frequently adopt a perivascular arrangement. We observed processes of telocytes near the mature adipocytes that form the lesion (Figure 9C,D), or immersed within fat released in the adipose tissue interstitium (Figure 9D) (original observation). Fat released between thick collagen fibres are also observed (Figure 9E).

### 5.3. TCs/CD34+SCs in Irradiated Adipose Tissue

Surgically resected rectal and perirectal tissues after preoperative radiotherapy for rectal cancer (Figure 9F) and irradiated tissues of the thymic region subsequently resected by other processes (Figure 9G,H) enabled our study of TC/CD34+SC behaviour in irradiated adipose tissue, not affected by cancer or other processes (unpublished observations). In both cases, TCs/CD34+SCs in the adipose tissue were increased in number and showed prominent processes, which extended around adipocytes (Figure 9F,G). In the rectum, the findings for TCs/CD34+SCs were similar in tissue components other than adipose tissue. Likewise, in the thymic region, TCs/CD34+SCs were observed separating thin strips of thymic epithelium (CKAE1/AE3+) from the adipocytes (Figure 9G,H). Therefore, exposure to ionising radiation has a hyperplastic and hypertrophic effect on TCs/CD34+SCs present in different tissues, including fat.

## 6. General Considerations about the Behaviour of TCs/CD34+SCs According to the Pathological Process in Adipose Tissue, Conclusions and Required Future Studies

Throughout this work, we reviewed TCs/CD34+SCs in pathological processes affecting adipose tissue. Our contributions can be systematised as follows: (a) demonstration (original report) of TCs/CD34+SCs and their characteristics in insulin-amyloid type amyloidosis, signet ring cell carcinoma with a Krukenberg tumour and peritoneal dissemination (see below), lipomatous hypertrophy of interatrial septum, nevus lipomatosus superficialis of Hoffman–Zurhelle and irradiated adipose tissue, (b) confirmation of the presence of TCs/CD34+SCs and description of their specific characteristics in early stages of inflammation and repair through granulation tissue (see below), neuromas and hyperplastic neurogenic processes and in certain tumours/pseudo-tumours of adipose tissue and (c) confirmation of absence of TCs/CD34+SCs and presence of αSMA+ stromal cells in advanced stages of inflammation and repair through granulation tissue and in the stroma of the adenocarcinoma of the colon.

The characteristics and behaviour of TCs/CD34+SCs in the pathologic processes studied, in which these cells can be conserved, increased in number or lost, and/or modified in shape, size, immunomarker expression and arrangement, contribute to supporting several roles hypothesised for TCs. Thus, the observations suggest potential roles such as (a) precursor cells in inflammation and repair (see below) and in tumour stroma (e.g., colon adenocarcinoma); (b) critical regulators of local homeostasis (e.g., limiting amyloid spread in insulin-amyloid type amyloidosis); (c) intercellular communication by cell-to-cell signalling or extracellular shedding vesicles; (d) behaviour as nurse or limiting tumour cells (e.g., in signet ring carcinoma with Krukenberg tumour and peritoneal dissemination (see below); and (e) contributors in several adipose tumours of an important CD34+ cell population, which can participate in the production of the myxoid extracellular component present in some of these tumours. Further studies are required in these fields and above all by transmission electron microscopy.

Two highly interesting issues observed in TCs/CD34+SCs are the changes in CD34 expression and their presence around neoplastic cells. Indeed, when activated, TCs/CD34+SCs can conserve or lose CD34 expression. Likewise, this activation may be with or without a gain of αSMA expression. Repair through granulation tissue and the stromal reaction (repair that does not stop) in adenocarcinoma of the colon are examples of processes that affect adipose tissue with a loss of CD34 expression in TCs/CD34+SCs or the disappearance of these cells. The demonstration of mitoses and a high proliferative index in TCs/CD34+SCs, as well as CD34 and αSMA co-expression in early stages of inflammatory/repair processes, suggests a change in immunomarker expression (in this case, loss of CD34 and gain of αSMA) rather than the degeneration or disappearance of these cells. Similar findings occurring in cultured adipose-derived stromal cells strengthen this possibility. Conversely, the stromal reaction in nerve components, neuroglial units or certain neoplasms growing in adipose tissue occurs along with the conservation of CD34 expression in TCs/CD34+SCs.

An intriguing fact that may be of interest in the behaviour of certain neoplasms is the presence of carcinomatous cells surrounded by TCs/CD34+SCs (e.g., in signet ring carcinoma with Krukenberg tumour and peritoneal dissemination). Indeed, this original observation raises the question of whether TCs/CD34+SCs act as nurse and/or insulating cells in specific types of tumours.

Two progenitor subsets of CD34+ cells (TCs/CD34+SCs) in lobules of adipose tissue have recently been described depending on septal or intralobular location [18]. CD34+ cells in fibrous septa are predominant myofibroblast precursors, while intralobular CD34+ cells have adipogenic capacity [18]. Different TC/CD34+SC behaviour in repair has also been described depending on CD34 location [26], an issue that requires further study.

In conclusion, we presented examples of TCs/CD34+SCs in different processes affecting the adipose tissue, including inflammation and repair through granulation tissue, iatrogenic insulin-amyloid type amyloidosis, non-adipose tissue components (nerve fascicles and fibres in neuromas and hyperplastic neurogenic processes) and tumours (signet ring carcinoma with Krukenberg tumour and colon carcinoma) growing in the adipose tissue, adipose tissue tumours (spindle cell lipoma, dendritic fibromyxolipoma, pleomorphic lipoma, infiltrating angiolipoma of skeletal muscle and elastofibrolipoma), lipomatous hypertrophy of the interatrial septum, nevus lipomatosus cutaneous superficialis of Hoffman–Zurhelle and irradiated adipose tissue of perirectal and thymic regions. The findings in this study support the hypothesis about the roles of TCs/CD34+SCs in homeostasis of the local microenvironment, intercellular communication and as progenitor cells. New studies using transmission electron microscopy are required and on the behaviour of TCs/CD34+SCs in other pathologic processes of the adipose tissue, specifically in lipomatosis and obesity.
